# How Do Muscle Function and Quality Affect the Progression of KOA? A Narrative Review

**DOI:** 10.1111/os.14022

**Published:** 2024-03-04

**Authors:** Lei Chen, Haojing Zhou, Yichen Gong, Yi Tang, Hai Su, Zhaokai Jin, Guoqian Chen, Peijian Tong

**Affiliations:** ^1^ The First Affiliated Hospital of Zhejiang Chinese Medical University (Zhejiang Provincial Hospital of Chinese Medicine) The First Clinical College of Zhejiang Chinese Medical University Hangzhou China; ^2^ Department of Orthopaedics and Traumatology The First Affiliated Hospital of Zhejiang Chinese Medical University Hangzhou China

**Keywords:** Muscle knee osteoarthritis, Muscle strength, Muscle, Knee osteoarthritis, Muscle cross section area

## Abstract

Knee osteoarthritis (KOA) is widely recognized as a chronic joint disease characterized by degeneration of knee cartilage and subsequent bone hyperplasia. However, it is important to acknowledge the significant role of muscles in the development and progression of KOA. Muscle function (MF) and muscle quality (MQ) are key factors in understanding the involvement of muscles in KOA. Quantitative indices such as muscle mass, muscle strength, muscle cross‐sectional area, muscle thickness, and muscle fatigue are crucial in assessing MF and MQ. Despite the growing interest in KOA, there is a scarcity of studies investigating the relationship between muscles and this condition. This review aims to examine the commonly used indices and measurement methods for assessing MF and MQ in clinical settings, while also exploring the association between muscles and KOA. Furthermore, this article highlights the importance of restoring MF and MQ to enhance symptom management and improve the quality of life for patients with KOA.

## Graphical abstract

Muscles around the knee.

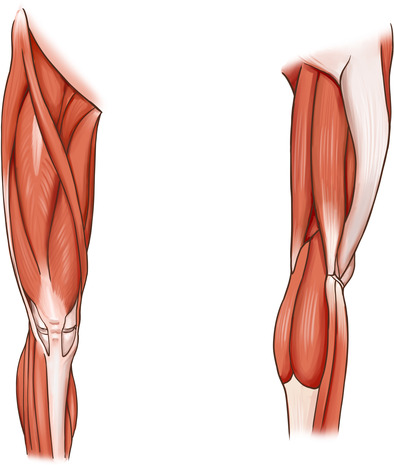

## Background

Knee osteoarthritis (KOA) is a degenerative condition affecting the structures around the knee joint, and its incidence and prevalence increase with age. The American College of Rheumatology classifies KOA into two main types: symptomatic knee osteoarthritis (SKOA) and radiographic knee osteoarthritis (RKOA).^1^ The development of KOA involves a complex interplay of biological and mechanical factors, resulting in various pathological changes, including cartilage loss, cartilage destruction and fissures, subchondral osteosclerosis, weakening and laxity of patellar‐related muscles and ligaments, meniscus wear and tear, and synovial and fat pad lesions.^2^, ^3^ Notably, the deterioration of muscle function (MF) and muscle quality (MQ) is a significant pathological aspect of KOA. Skeletal muscles, serving as crucial components of the motor system, play a vital role in joint function and movement. MF and MQ are closely linked to the onset, progression, and symptoms of KOA. For instance, reduced quadriceps strength can exacerbate knee pain in patients with KOA and contribute to disease progression.^4^, ^5^ Additionally, diminished strength in the knee extensors and flexors increases the risk of deterioration in tibiofemoral arthritis and patellofemoral arthritis.^6^ Elderly individuals aged 70–80 with the lowest muscle density quartile faced a 51% higher risk of hospitalization compared to those in the highest quartile.^7^ Conversely, the restoration of MF and MQ can alleviate pain, improve joint function, and enhance the quality of life for individuals with KOA.^5^, ^8^

Currently, several clinical assessment indices are available for evaluating MF and MQ in the knee area. These indices include muscle strength, muscle mass, muscle cross‐sectional area (CSA), muscle thickness, muscle fatigue, among others. This article aims to provide a comprehensive review of the common factors and measurement methods used to assess MF and MQ. Additionally, it will explore the relationship between muscle and KOA. The article will emphasize the importance of restoring MF and MQ to improve symptoms and enhance the quality of life for patients with KOA. To ensure comprehensive coverage, systematic reviews and relevant investigations have been incorporated into the study to minimize the possibility of omitting crucial research findings.

## Muscle Strength

### Measurement of Muscle Strength

Muscle strength is vital for knee stability and function. Isokinetic dynamometry, a classic method for measuring muscle strength, has been reliably used for over 40 years. It enables evaluation of maximal strength at a constant speed, including isometric, concentric, and eccentric strength.^9^, ^10^, ^11^ While isokinetic dynamometry provides reliable data for knee extension in specific populations, its widespread clinical use is limited due to high costs. Additionally, the standard error of measurement varies among authors when assessing lower limb strength.^12^ Recent studies suggest that hand‐held dynamometry is a reliable and valid alternative to isokinetic dynamometry, serving as a supplement.^13^, ^14^

Recent technological advancements have significantly improved muscle strength measurement instruments. These upgrades encompass stretchable, self‐healing, and skin‐mounted active sensors, isometric myodynamia and real‐time rotation sensing systems, etc. These innovations enhance portability, measurement precision, and functionality compared to previous models, offering more practical tools for clinical research. These advancements aim to yield more clinically meaningful outcomes for KOA patients.^15^, ^16^

These cutting‐edge instruments enable more precise and reliable evaluations of muscle strength in KOA patients. They facilitate detailed assessments, allowing researchers to accurately track changes in muscle strength, assess damage extent, and monitor rehabilitation progress. This information is vital for refining treatment and rehabilitation strategies for KOA patients.

### How Does Muscle Strength Affect KOA?

Currently, most studies indicate that muscle strength is closely related to pain and the weakening of the muscle strength in thigh muscle group especially. In a study, by Øiestad *et al*.^17^ it was found that the weakening of extensor muscles accelerated the development of SKOA in both males and females. However, Segal *et al*.^18^ measured thigh strength in 5911 knees, of which 2519 were SKOA knees and 3392 were RKOA knees. The results indicated that decreased thigh muscle strength predicted the occurrence of SKOA but did not show a significant correlation with RKOA. Other studies have also suggested that both SKOA and RKOA are closely associated with reduced strength in the extensor or flexor muscles (see for instance, Slemenda *et al*).^19^ Other studies have also suggested that both SKOA and RKOA are closely associated with reduced strength in the extensor or flexor muscles. While Culvenor *et al*.^20^ demonstrated that female RKOA patients exhibited relatively reduced strength in the extensor and flexor muscles compared to healthy individuals, while there was no significant change observed in male RKOA patients.

Among the periarticular muscles of the knee, the quadriceps plays a crucial role in stabilizing the knee and providing cushioning during walking.^21^, ^22^ Studies have investigated the impact of the quadriceps muscle on KOA. It is believed that weakened quadriceps muscle strength impairs its ability to control tibial movement during walking, increasing the risk of structural knee injuries. Additionally, the reduced cushioning effect of the quadriceps raises intraarticular pressure and contributes to joint damage.^23^ Some studies suggest that weakened quadriceps muscle strength is more likely to cause muscle fatigue, which affects the proprioception of the knee joint and leads to neuromuscular disorders, thereby impacting joint stability.^24^, ^25^, ^26^ Moreover, there is substantial evidence suggesting that hip muscle weakness contributes to lower extremity conditions.^27^, ^28^, ^29^ It is proposed that weakened hip muscles can increase medial compartment loading on the knee joint. While there is strong evidence recommending hip muscle strengthening in the conservative management of individuals with KOA, further research is needed to establish underlying mechanisms and clinical benefits.^30^ Regarding the knee flexor muscles, they are not as influential as the quadriceps in the development of KOA. However, some researchers have found that low knee flexor strength is associated with an increased risk of worsening tibiofemoral osteoarthritis. Knee flexor function is considered important for knee joint health in individuals with or at risk of KOA.^6^ Wild *et al*.^31^ suggested that decreased hamstring muscle strength leads to misalignment of the frontal plane of the knee joint in KOA patients, compromising the control of knee joint alignment and increasing the load on the knee, thus elevating the risk of KOA. The knee flexors are increasingly recognized as having a significant impact on knee kinematics and kinetics.^32^

Within current clinical practice, there exists a widespread acknowledgment regarding the pivotal importance of muscular strength in individuals afflicted by KOA. A spectrum of therapeutic strategies has emerged from this recognition. These encompass methodologies such as isokinetic exercises^33^ and a combination of nutritional interventions with resistance training,^34^ all directed toward augmenting the muscular strength of KOA patients, thereby aiming to alleviate their symptoms. Concurrently, a myriad of research articles has highlighted the pervasive nature of muscular strength deficiencies observed among KOA patients, regardless of the severity of their individual conditions.

Upon undergoing tailored and specialized muscular strength training, these patients exhibited notable enhancements in muscular strength, reduction in pain levels, mitigation of depressive symptoms, and improved stability of the knee joint.^35^, ^36^ Notably, this intervention also yielded a significant deceleration in the progression of knee osteoarthritis. This unequivocally emphasizes the critical role that muscular strength plays in the advancement of patellofemoral osteoarthritis.^37^, ^38^

In general, muscle strength is considered a vital marker closely associated with KOA. Despite abundant research substantiating the significance of muscle strength in KOA, its precise role within the pathophysiological mechanisms of KOA remains inadequately elucidated. Consequently, further in‐depth investigations are warranted to delineate the impact of muscle strength on KOA. Nevertheless, this does not impede the clinical application of various methodologies, such as resistance training and blood flow restriction training, to effectively enhance muscle strength, ameliorate KOA symptoms, and slow the progression of the disease.

## Muscle Mass

### Measurement of Muscle Mass

Muscle mass refers to the proportion of muscle tissue within the body. It is essential for the proper functioning of the human body. Insufficient muscle mass, such as in cases of muscular atrophy or sarcopenia, can negatively impact normal physiological muscle function. Conversely, pathological increases in muscle mass can lead to muscular hypertrophy. Maintaining an appropriate amount of muscle mass is crucial for optimal physiological function.^39^, ^40^

In clinical settings, there are four primary methods commonly used to assess muscle mass: bioelectric impedance analysis (BIA), computed tomography (CT), dual‐energy X‐ray absorptiometry (DXA), and magnetic resonance imaging (MRI). BIA is a non‐invasive technique that measures the impedance of electrical currents passing through the body to estimate body composition, including muscle mass. DXA, a widely used method, employs X‐ray technology to measure bone density and body composition, including muscle mass. CT scans provide detailed cross‐sectional images of the body, allowing for the quantification of muscle tissue. Similarly, MRI utilizes powerful magnets and radio waves to generate images that can be used to assess muscle mass. Comparisons show BIA's satisfactory accuracy for fat‐free mass against DXA (*r* ^2^ = 0.6275, *p* < 0.0001) and CT (*r* ^2^ = 0.274, *p* < 0.0001), particularly in clinical and research contexts.^41^, ^42^ In studies with healthy individuals, BIA assessments demonstrated 3–5% accuracy compared to gold standard methods like air‐displacement plethysmography, DXA, MRI, and CT.^43^ These four methods offer valuable tools for evaluating muscle mass in clinical practice, providing insights into the quantity and distribution of muscle tissue.^44^, ^45^

### How Does Muscle Mass Affect KOA?

Recent reports have highlighted the association between muscle mass and the progression and clinical symptoms of KOA. Jeon *et al*.^46^ conducted a cross‐sectional study that demonstrated an independent association between muscle mass and RKOA. Suh *et al*.^47^ found that lower limb muscle mass was linked to the occurrence and severity of KOA in women. Park *et al*.^48^ reported that muscle mass in the lower limbs was a risk factor for knee pain in patients with mild RKOA, but no significant correlation was observed in severe RKOA patients. Similarly, Lee *et al*.^49^ concluded that lower muscle mass was associated with joint pain in KOA patients with a Kellgren–Lawrence level of ≥2. These studies collectively suggest that lower muscle mass is significantly correlated with knee pain in KOA patients, and the correlation may vary based on gender and the Kellgren–Lawrence level. However, it is important to note that some studies have reported conflicting findings regarding the association between muscle mass and KOA. Research comparing muscle mass between early and late‐stage KOA patients has revealed that the muscle mass of vastus medialis and vastus intermedius is typically higher in early‐stage patients compared to late‐stage individuals.^50^ Segal *et al*.^51^ found no apparent association between thigh muscle mass and SKOA, RKOA, or joint space stenosis. The discrepancy in these findings may be attributed to the failure to account for factors such as age and weight in the data analysis.^52^

Some studies have investigated the potential relationship between decreased lower limb muscle mass and instability and pain in the knee joint. They propose that a reduction in lower limb muscle mass could contribute to knee joint instability and pain, potentially promoting the progression of KOA and the onset of symptoms.^49^ However, it is important to note that another study suggests a different perspective. This study proposes that muscle strength and lower extremity muscle mass are independent factors and do not directly affect each other. According to this research, impaired nerve activation caused by KOA primarily affects lower extremity muscle strength rather than muscle mass.^53^

The findings suggest that a decrease in muscle mass may exacerbate symptoms of KOA such as pain, joint instability, and reduced gait speed. Specific clinical investigations have observed a reduction in muscle mass in certain areas among severe KOA patients compared to those with mild KOA. Currently, there remains a lack of definitive literature elucidating how changes in muscle mass affect the progression of KOA and its clinical symptoms. However, the intricate relationship between muscle mass and muscle strength complicates this understanding, with neurological impairments associated with KOA potentially exerting a greater influence on muscle strength than on muscle mass. Therefore, further high‐quality, meticulously designed studies are required to establish the interplay between muscle mass, muscle strength, and KOA. Nevertheless, increasing muscle mass appears to contribute to managing the clinical symptoms and progression of KOA to some extent.

Consequently, the author encourages healthcare professionals to more extensively adopt nuanced approaches in clinical practice. This entails enhancing muscle mass among KOA patients to alleviate their symptoms. Moreover, tailoring clinical strategies based on varying levels of muscle mass in KOA patients at different stages is advocated for optimal care.

## Muscle Cross‐sectional Area

### Measurement of Muscle Cross‐sectional Area

CSA is recognized as a significant indicator of muscle mass, with its increase or decrease directly impacting muscle function.^54^, ^55^ Various methods exist to assess muscle CSA, including hands‐free circumference measurement, ultrasound techniques, CT, and MRI.^56^, ^57^ Ultrasound techniques have emerged as the preferred clinical option due to their portability, affordability, and safety for repeated measurements.^43^, ^58^, ^59^ While MRI and CT offer high precision and resolution with accurate qualitative and quantitative data, they are not commonly chosen due to their immobility, high cost, and radiation associated with CT.^60^, ^61^

### How Does Muscle Cross‐sectional Area Affect KOA?

Regarding the association between knee muscle CSA and KOA, longitudinal studies have shown that CSA changes occur more rapidly in thigh muscles than changes in muscle strength in adult patients.^62^, ^63^ Yamauchi *et al*.^64^ used MRI and found that intermediate and advanced KOA patients had greater CSA in the vastus medialis, vastus lateralis, and vastus intermedius compared to early KOA patients. Vastus intermedius CSA was also associated with the Kellgren–Lawrence level. Meanwhile, in other studies, it has been found that after training, there is an increase in the muscle cross‐sectional area (muscle CSA) among early‐stage patients. This increase is associated with reduced intra‐articular pressure and improvements in symptoms such as pain.^65^ Similarly, Silva *et al*.^66^ observed a 10% reduction in gastrocnemius CSA in KOA rats compared to normal rats, suggesting the importance of maintaining proper extensor and flexor balance to slow patellofemoral cartilage deterioration. However, there are contrasting viewpoints regarding the association between quadriceps muscle CSA and RKOA.^67^ There are other thoughts that quadriceps muscle CSA has no association with RKOA.^55^, ^68^ Ruhdorfer *et al*.^55^ conducted longitudinal studies and found no significant change in quadriceps CSA in the lower limbs of patients with early unilateral KOA. It is worth noting that knee pain and KOA can cause muscle atrophy^66^, ^67^ leading to changes in muscle mass and affecting normal muscle function.^39^, ^40^, ^68^, ^69^ Furthermore, some studies suggest that muscle CSA may not be directly related to KOA but can influence its progression by affecting muscle strength, mass, and other related factors.^70^, ^71^

Relatively speaking, the association between muscle CSA and KOA is not as closely linked as the relationship between muscle strength, muscle mass, and KOA. Moreover, there is insufficient high‐quality research supporting the direct and independent impact of muscle CSA on the progression and symptoms of KOA. This limitation might be attributed to the relatively low precision of measurement tools or insufficient ease of measurement for muscle CSA. If clinical instruments for measuring muscle CSA advance further, clinicians may develop a deeper appreciation for the significance of muscle CSA. In summary, the relationship between muscle CSA and KOA is multifactorial, necessitating further clinical and foundational experiments to elucidate this association.

## Other Indexes

### Muscle Thickness

Muscle thickness is one of the morphology indexes to describe the muscle. Ultrasound techniques, CT and MRI are widely used to measure muscle thickness in the clinic. Ultrasound techniques are a safe, reliable, and simple way to measure muscle thickness. To a certain degree, they can reflect muscle CSA.^68^, ^72^ Koca *et al*.^73^ indicated that muscle thickness was negatively correlated with Kellgren–Lawrence level in KOA patients. However, due to the small sample size in this study, larger sample sizes and multicenter studies are needed to support the theory of the impact of muscle thickness on KOA.

### Muscle Fatigue

Muscle fatigue is an evaluation index of muscle. It is typically defined as the decline in maximal contractility caused by exercise. It may be associated with peripheral nerve changes or inadequate activation of motor neurons by central neurons.^74^ Currently, muscle fatigue is primarily assessed using surface electromyography (EMG). Mau‐Moeller *et al*.^75^ conducted an EMG study involving 20 KOA patients and 20 healthy individuals, and they found that KOA patients' muscles were more prone to fatigue. Watanabe *et al*.^76^ examined neuromuscular activation of the femoris intermedius during fatiguing muscle contractions in seven healthy men using electromyography. They observed inconsistent neuromuscular activation patterns across different muscles during negative maximum contractions. However, it should be noted that a study cautioned against using surface EMG to assess fatigue during multi‐joint sub‐maximal isometric quadriceps testing due to poor between‐day reliability and high measurement error.^77^

### Mechanical Property and Muscle Asymmetry

Except for the ones discussed, MyotonPRO invented by researchers in Estonia is used to examine the mechanical property of muscles.^78^ Lee *et al*.^79^ also found that leg muscle asymmetry in men was associated with a significantly higher grade of radiographic knee OA and prevalent knee pain. The relationship between these factors and KOA is worthy of further research.

While quantifiable indicators of other MF and MQ, such as muscle thickness, muscle fatigue, and muscle symmetry, have not exhibited a significant correlation with KOA, clinical practitioners have been able to discern potential impacts of these indicators on KOA patients within nuanced aspects. However, constrained by insufficient emphasis on these indicators and low adaptation of measurement tools, the clinical field has yet to yield high‐quality, reliable research articles substantiating the precise relationship between these indicators and KOA. We aspire that alongside strengthening the detection of these indicators, clinical practitioners will harness these metrics to ameliorate symptoms in KOA patients and effectively impede disease progression. This advancement holds promise in furnishing more robust evidence for future research and therapeutics to better manage and address the condition of patellofemoral osteoarthritis.

## Restoring Muscle Function and Quality

The existing research indicates a potential association between the decline of MF and MQ and the development of KOA. Specifically, decreased quadriceps muscle strength and lower limb muscle mass have been identified as independent risk factors for KOA. Clinical guidelines for KOA patients also emphasize the importance of restoring MF and MQ to alleviate symptoms.^80^, ^81^ Therefore, interventions aimed at restoring MF and MQ hold promise in the prevention and treatment of KOA.

### Exercise Therapy

Exercise therapy is a crucial intervention for improving symptoms and restoring MF and MQ in individuals with KOA. Various exercise modalities, including Tai Chi, yoga, aquatic exercise, and others, have been investigated in relation to KOA and have shown efficacy in improving symptoms, muscle performance, and various muscle indicators in KOA patients^82^, ^83^, ^84^, ^85^, ^86^ Lee *et al*.^87^ demonstrated that Tai Chi could alleviate pain and enhance muscle strength in the flexor and extensor muscles of KOA patients. Bruce‐Brand *et al*.^88^ also found that exercise could increase the CSA of quadriceps. These observed improvements may be attributed to the capacity of muscle training to induce structural and functional adaptations in skeletal muscle cells.

### Physical Therapy

Physical therapies, apart from exercise therapy, can effectively alleviate symptoms in KOA patients. Knee taping, a recommended physiotherapy strategy, has shown promising results in reducing pain by alleviating stress on the patellofemoral joint and relieving discomfort in soft tissues surrounding the knee joint. Numerous randomized controlled trials have demonstrated immediate and short‐term pain reduction through knee taping in patients with knee OA, regardless of patellofemoral joint involvement. This approach holds potential for managing knee OA and improving the overall well‐being of affected individuals.^89^, ^90^, ^91^ With or without muscle‐strengthening exercises, neuromuscular electrical stimulation, transcutaneous electrical nerve stimulation, and ultrasound therapy have advantages for muscle strength, structure, and pain in KOA patients, in addition to improving motor capacity and quality of life.^92^, ^93^, ^94^, ^95^

### Manual Therapy

Manual therapy also takes a large part in restoring MF and MQ. Kaya Mutlu *et al*.^93^ have found that after manual therapy, terms of pain level, knee range of motion, quadriceps muscle strength, and functional level were improved. Tsokanos *et al*.^96^ also have a similar conclusion that manual therapy can contribute positively to the treatment of patients with KOA by reducing pain and increasing functionality. As for Chinese traditional manual therapy—Tuina is widely used in the clinic and has proven effective in KOA patients. Zheng *et al*.^97^ indicated that Tuina may benefit in improving muscle tone, blood circulation, flexibility, and inhibition of inflammatory factors. Tuina combined with acupuncture could improve the clinical therapeutic effect of patients with knee osteoarthritis.^98^

### Diet

Diet is one of the effective ways to prevent and treat KOA as well. On the one hand, low‐salt and low‐fat diet can lose weight which can be concluded as one of the pathogenic factors of KOA. On the other hand, a nutritious diet may also improve muscle function and mass, effectively slowing muscle mass loss and preventing muscle strength loss. Henriksen *et al*.^99^ indicate that control and reasonable diet can significantly reduce the weight of obese patients, lower limb fat, and standard muscle strength increase by 11–12%.

### Traditional Chinese Medicine

Traditional Chinese Medicine also plays a unique role in restoring muscle function and quality. Zhang *et al*.'s^100^ study found that Icaritin, the active component of Epimedium, can counteract skeletal muscle atrophy following mechanical unloading through PI3K/Akt signaling in Sprague–Dawley rat models. Oh *et al*.^101^ also found that newly isolated flavonoid and 5‐aminopyridine in the Chinese chive (Allium tuberosum) up‐regulated PI3K/Akt/mTOR pathways, which implies a positive effect on skeletal muscle growth and differentiation and suggests that major constituents of Chinese chive, flavonoids, and amino acids, might be used in dietary supplements that aid skeletal muscle growth.

### Toal Knee Arthroplasty

Total knee arthroplasty (TKA) is the main surgical method for severe KOA patients. MF and MQ are greatly reduced after surgery.^102^, ^103^, ^104^ A great number of patients suffer from reduced exercise capacity Chughtai *et al*.^105^ study that multiple methods such as neuromodulation cooling therapies and compression are used to reduce pain and improve muscle strength. Klika *et al*.^106^ also found neuromuscular electrical stimulation is a good way to support a quicker return to function after TKA. No matter Preoperatively or postoperatively, MF and MQ are vital to KOA patients.

## Conclusion

The degeneration of muscle strength, muscle mass, and muscle CSA, among other factors, signifies prominent pathological characteristics associated with KOA. The deterioration in muscle function and quality intricately correlates with the onset and progression of KOA. Enhancing muscle strength not only mitigates knee osteoarthritis symptoms but also contributes to maintaining joint stability and reducing pain. Sufficient muscle mass offers support and safeguards for KOA patients, aiding in diminishing joint pressure and decelerating disease advancement.

While factors such as muscle CSA, muscle thickness, and muscle fatigue might not independently dictate KOA, they significantly influence the disease's progression through complex mechanisms. The correlation between KOA and MF and MQ is multifaceted, necessitating extensive clinical and foundational research for comprehensive exploration. To explicitly delineate the interrelation among measurement tools, areas, and indicators, a dedicated Table 1 has been meticulously constructed.

**TABLE 1:** Muscle and measurement.

MF and MQ quantive indexes	Meassure methods	Location	Detection index
Muscle strength	Isokinetic dynamometer	Knee extensor and flexor^17^, ^18^, ^22^, ^33^	Flexor and extensor torque Concentric and eccentric strength
	Good strength chair	Knee extensor and Flexor^20^, ^63^	Thigh muscle anatomical cross‐sectional areas
	Bicycle ergometer	Quadriceps^26^, ^31^	Functional mobility, knee joint Proprioception, quadriceps force accuracy and steadiness, and maximal quadriceps strength
	Handheld dynamometer	Upper limbs^14^	Muscle quality index (handgrip strength divided by lean arm mass)
Mascle mass	DEXA	The whole body and both lower extremities^46^, ^47^, ^48^, ^49^, ^52^	Appendicular skeletal muscle mass Percentage of muscle mass in both lower extremities Lean mass of whole body and both legs
	Ultrasound	Vastus medialis, biceps femoris, gluteus maximus, gluteus medius, gastrocnemius, soleus and tibialis anterior rectus femoris, vastus lateralis and vastus intermedius^50^	Echo intensity
	Densitometer	Thigh^51^	Thigh lean body mass
Muscle cross‐section areas	MRI	10 cm proximal to the distal femoral epiphysis, extending 7.5 cm proximally quadriceps hamstrings and adductors^62^, ^63^, ^67^ Rectus femoris, vastus lateralis, vastus intermedius, vastus medialis, biceps femoris short head, biceps femoris long head, semitendinosus, semimembranosus, adductor, sartorius, and gracilis.^64^, ^107^ Flexor and extensor^70^	Muscle cross‐section areas
	CT	Rectus femoris, vastus lateralis, vastus intermedius, vastus medialis^71^	Muscle cross‐section areas
Other indexes	Ultrasound	Ntermedius and rectus femoris from the points (a) at the border between the upper and lower two‐thirds and point (b) at the center of the line between the anterior superior iliac Spine and the upper pole of the patella.^73^	Muscle thickness
	Electromyogram	Quadriceps^75^	Isometric submaximal fatiguing voluntary contraction
		Rectus femoris, vastus lateralis, vastus medialis and vastus intermedius^76^	Surface EMGs from the rectus femoris, vastus lateralis, vastus medialis and vastus intermedius
	MyotonPRO	Quadriceps^78^	The mechanical property of muscles
	DEXA	Both legs^79^	Leg muscle asymmetry

Abbreviations: CT, computed tomography; DEXA, dualenergy X‐ray absorptiometry; MF, muscle function; MQ, muscle quality; MRI, magnetic resonance imaging.

Various measurement methods, such as CT, MRI, and DXA for assessing muscle mass and muscle CSA, and isokinetic strength testing for evaluating muscle strength, cater to different muscle quantification indicators. Each measurement tool possesses distinct advantages and is applicable in different scenarios. Nevertheless, for indicators like muscle CSA, muscle thickness, and muscle fatigue, exploring more suitable and precise measuring instruments is essential for evaluating MF and MQ.

MF and MQ exhibit close associations with KOA, with muscle weakness and diminished quality identified as potential KOA risk factors. Despite an incomplete understanding of the precise correlation between muscle strength, mass, CSA representing MF and MQ, and KOA, interventions directed toward MF and MQ, such as exercise therapy, physical treatments, and dietary adjustments, have demonstrated notable efficacy in alleviating KOA symptoms, enhancing muscle performance, and reinstating MF and MQ. Furthermore, the notable decline in MF and MQ, TKA has prompted significant attention regarding muscle status both pre‐ and post‐surgery. Within clinical practice, numerous researchers have effectively improved diverse measurable indicators of MF and MQ through methodologies encompassing isokinetic strength training, resistance training, and blood flow restriction training, thereby mitigating KOA‐related symptoms. This comprehensive review seeks to analyze thoroughly the relationship between various parameters and KOA, underscoring their importance in KOA patient management. The overarching objective is to offer guidance to clinical practitioners for employing muscle‐focused interventions in the management of KOA symptoms, with the ultimate aim of retarding the progression of KOA.

In summary, the relationship between MF and MQ and KOA is intricate and cannot be disregarded. Rehabilitating muscle function and quality is crucial for improving symptoms and quality of life for KOA patients. Effective muscle training and rehabilitation measures enhance muscle function and quality, alleviate joint burden, slow disease progression, and enhance patient quality of life. Future research should prioritize intervention strategies targeting muscle strength and quality, determining optimal rehabilitation regimens, and offering more effective treatment strategies.

## Conflict of Interest Statement

The authors stated that they had no interests which might be perceived as posing a conflict or bias.

## Author Contributions

All authors contributed to the study conception and design. The basic framework and conception of this article were performed by Lei Chen and Haojing Zhou. The first draft of the manuscript was written by Lei Chen, and all authors commented on previous versions of the manuscript. All authors read and approved the final manuscript.
